# Professional Activity and Cognitive Performance in Schizophrenia

**DOI:** 10.1192/j.eurpsy.2026.12147

**Published:** 2026-07-31

**Authors:** D. Njah, M. Lagha, A. Zyed, W. Homri

**Affiliations:** Psychiatry c, Razi psychiatric hospital, Manouba, Tunisia

## Abstract

**Introduction:**

Cognitive dysfunction is a central feature of schizophrenia and contributes significantly to functional disability. It affects memory, attention, executive functions, and processing speed, thereby impacting daily life and social integration. Determining factors that contribute to preserving cognitive function is therefore essential. Professional activity appears to play a beneficial role by providing continuous cognitive stimulation, structuring routines, and supporting patients’ social engagement.

**Objectives:**

Our objective was to assess the impact of professional activity on cognitive function in patients with schizophrenia and schizoaffective disorder (SAD).

**Methods:**

This was a cross-sectional study including patients with schizophrenia and SAD, aged between 18 and 65 years, that took place over a 2-month period, from February 1, 2025 to April 15, 2025 who were followed up at RAZI psychiatric hospital in Tunisia. Cognitive function was assessed using the Arabic version of the Montreal Cognitive Assessment (MoCA) scale. Information regarding patients’ occupational status was obtained through interviews and verified using medical records.

**Results:**

We included 56 patients with schizophrenia and 14 patients with SAD. The mean age was 42 years (SD, 11). Sixty seven percent of our population were male(n=47) and almost 76% (n=53) were single. Among the patients, 24% (n=17) were professionally active, while 76% (n=53) were not. The median MoCA score was 20(interquartile range, 16-25 ; range 5-30). Cognitive dysfunction was found in 74,3% of our population(n=52). Professionally active patients had significantly higher MoCA scores than non-active patients (p = 0.002).

**Conclusions:**

Our findings suggest that professional activity may have a protective effect against cognitive dysfunction in patients with schizophrenia and SAD. Facilitating occupational engagement and professional integration should therefore be considered an essential component of comprehensive care for this population.

**Disclosure of Interest:**

None Declared

